# Cyclodextrin Induces Calcium-Dependent Lysosomal Exocytosis

**DOI:** 10.1371/journal.pone.0015054

**Published:** 2010-11-29

**Authors:** Fannie W. Chen, Chunlei Li, Yiannis A. Ioannou

**Affiliations:** Department of Genetics and Genomic Sciences, The Mount Sinai School of Medicine, New York, New York, United States of America; Biological Research Center of the Hungarian Academy of Sciences, Hungary

## Abstract

Cyclodextrins (CDs) have long been used to manipulate cellular cholesterol levels both *in vitro* and *in vivo*, but their direct effects at a cellular level are not well characterized. Recently, CDs have garnered much interest because of their ability to clear stored cholesterol from Niemann Pick Type C (NPC) cells and markedly prolong the life of NPC1 disease mice. Here, we investigate the hypothesis that treatment with 2-hydroxypropyl- β-cyclodextrin (HPB-CD) stimulates lysosomal exocytosis in a calcium-enhanced manner. We propose that this exocytosis is the mechanism by which HPB-CD ameliorates the endolysosomal cholesterol storage phenotype in NPC cells. These findings have significant implications for the use of HPB-CD in biochemical assays and data interpretation as well as for their use for the treatment for NPC and other disorders.

## Introduction

Beta-cyclodextrins (CDs) are a family of cyclic oligosaccharides composed of a hydrophilic outer surface and a hydrophobic core that has a high affinity for sterols [1]. Because of these characteristics, their use for the manipulation of cellular cholesterol content has been pervasive [2], although the precise mechanism of their actions has not been fully elucidated. They are presumed to disrupt cholesterol-rich membrane domains and sequester lipids within their core [3], but whether these actions are limited to the plasma membrane or extend to organellar membranes has not been established. A recent study showing that CDs also extract sphingomyelin from lipid bilayers [4] highlights the deficiency in our current knowledge regarding the actions of CD in a cellular milieu.

Although CDs have been used as formulation vehicles by which lipophilic drugs are incorporated into aqueous solutions, 2-hydroxypropyl-β-cyclodextrin (HPB-CD) has itself been recently approved for the treatment of Niemann Pick Type C (NPC) disease [5], which is characterized by a massive accumulation of lipids, including cholesterol, in the endosomal/lysosomal system. HPB-CD has produced extremely promising results in a mouse model of NPC1; it delays the onset of neurological symptoms and can prolong their lifespan by up to ∼120% [6], [7], [8]. Whether these results will be directly translatable in human trials remains to be determined. However, it is clear that questions regarding the mode of its action, as well as the consequences of prolonged exposure to the compound, are highly relevant for assessing the long-term effects of HPB-CD as a potential therapeutic intervention.

In these studies, we examine the effects of HPB-CD on cells derived from Wt and NPC1 mice. We show that exposure to HPB-CD stimulates lysosomal exocytosis, which is enhanced in the presence of extracellular calcium. During this process, endolysosomal components are extruded from cells, providing the basis for observations that exposure to HPB-CD rapidly clears NPC cells of stored cholesterol. To our knowledge, these findings represent the first direct characterization of the mechanism of HPB-CD action on cells, which have significant implications for both the use of HPB-CD as a treatment vehicle and data interpretation in experiments utilizing CD to manipulate cellular cholesterol levels.

## Results

### Exposure to cyclodextrin does not activate a stress-induced signaling response

**Figure 1:**
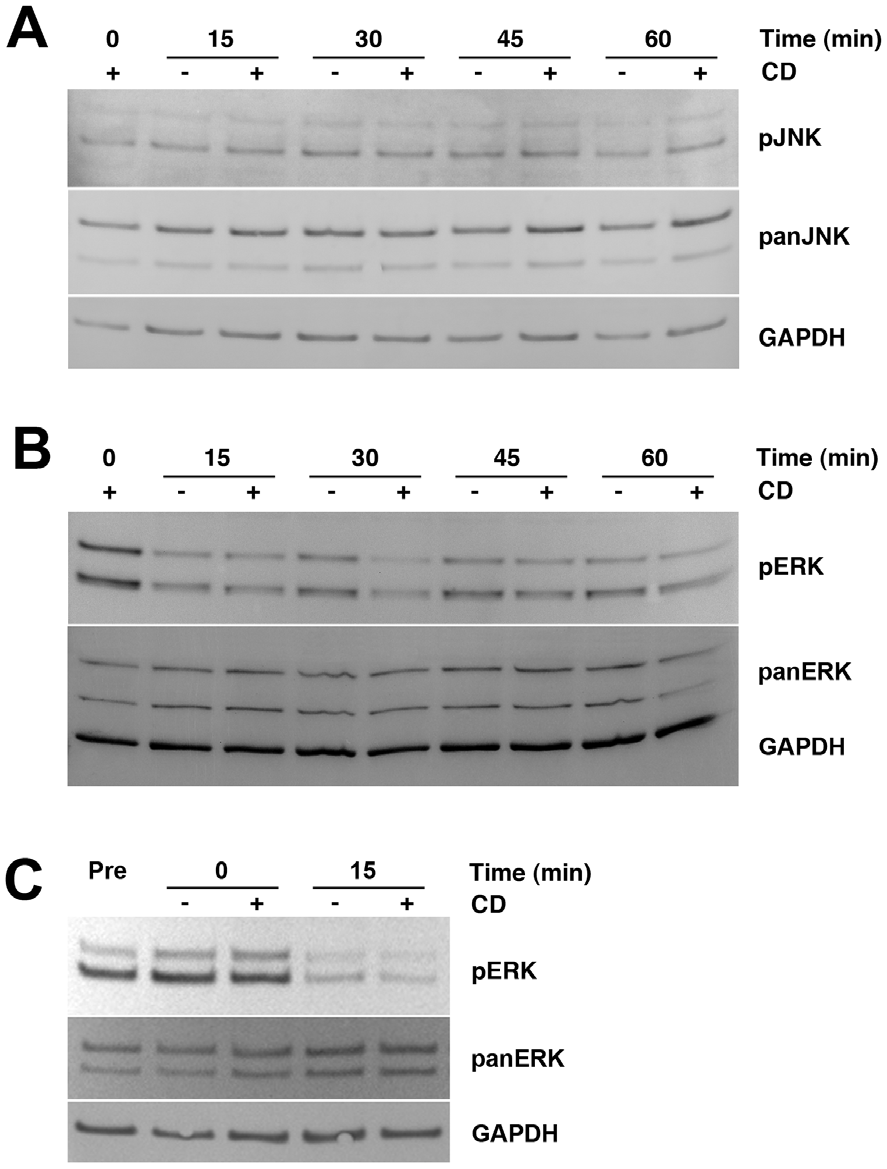
The effect of HPB-CD on stress-related protein kinases. Representative western blot analyses of total (pan) and phosphorylated (p) ERK (A) and JNK (B and C) in lysates from Wt cells treated with HPB-CD. The blots shown are representative of 3 independent experiments.

Cyclodextrins have long been used to extract plasma membrane cholesterol, which can result in depletion of up to half of the total cell cholesterol content within one hour of treatment [9]. Such a rapid change in plasma membrane cholesterol could trigger stress-induced signaling pathways, which could stimulate exosome release as has been previously described [10]. One such pathway is the stress-activated protein kinase/c-Jun N-terminal kinase (SAPK/JNK) pathway [11]. Also, cyclodextrins may activate the extracellular signal-regulated kinase (ERK) pathway; activated phosphorylated ERK has a multitude of targets involved in cell proliferation and survival. Cells derived from Wt BALB/c mice that were treated with HPB-CD for up to 1 hour exhibited no apparent increase in phosphorylated JNK (pJNK) or ERK (pERK) levels (Fig. 1). Immediately following HPB-CD treatment, there appears to be a slight increase in pERK (Fig. 1B, 0 min), but a comparison with pERK pretreatment levels or pERK levels immediately following treatment with vehicle alone did not reveal any changes in pERK levels immediately following treatment (Fig. 1C). The fact that the decreased levels of pERK between 0 and 15 minutes occur in both untreated and treated cells suggests that this particular response is not due to HPB-CD exposure but instead to the treatment conditions. Furthermore, the levels of the pan form of both kinases were also unchanged, indicating that these pathways are not affected by exposure to HPB-CD. These results indicate that the ability of HPB-CD to ameliorate the cholesterol storage phenotype in NPC mice is not due to activation of stress-induced signaling pathways.

### Exposure to cyclodextrin alters the cholesterol storage phenotype of NPC1 cells

**Figure 2:**
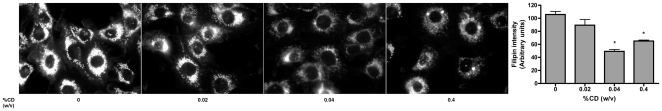
Filipin staining of free cholesterol in NPC1 cells treated with HPB-CD for 20 hours. Filipin intensity was quantitated in at least 150 cells for each sample. The bar graph represents average values from 3 independent experiments. *P<0.0001.

One of the biochemical hallmarks of NPC disease is the accumulation of free cholesterol in vesicles of the endosomal/lysosomal system, which can be visualized in cultured cells with the fluorescent antibiotic filipin. Since HPB-CD treatment reduces free cholesterol levels in various organs of NPC1 mice [6], [7], we first determined if the compound has a similar effect in cell lines derived from these NPC1 BALB/c mice. As expected, untreated cells (Fig 2, 0%) exhibit bright, punctate filipin staining that is indicative of accumulated free cholesterol in endocytic vesicles. The filipin intensity decreases as the HPB-CD concentration increases, indicating that exposure to the compound reduces the amount of stored cholesterol in these cells. Interestingly, at the highest concentration tested, the effectiveness of HPB-CD in clearing stored cholesterol appears somewhat decreased (Fig. 2, 0.4%). This result might be due to slight toxicity effects from overnight incubation with the compound, which has previously been reported in cultured cells [12].

**Figure 3:**
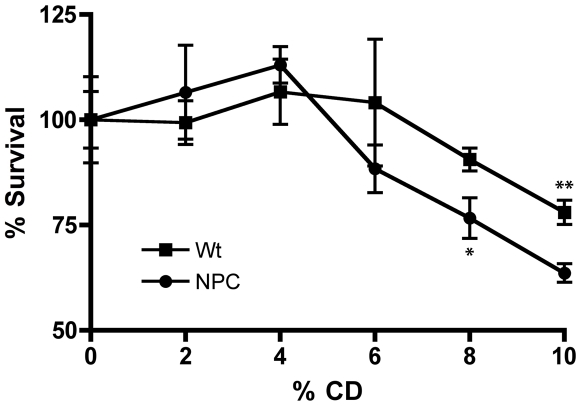
HPB-CD toxicity in cell lines derived from BALB/c mice. Wt (squares) and NPC1 (circles) cells were treated with increasing concentrations of HPB-CD for 2 hours and their percent survival was determined using the MTT assay as described in “Materials and Methods”. * and ** denote statistically significant differences between treated and untreated cells with *P*<0.01 and *P*<0.05, respectively.

Because the effects of HPB-CD on cholesterol metabolism have been shown to occur very rapidly in cultured cells [12], we next determined the toxicity threshold for acute HPB-CD treatment in cell lines derived from Wt and NPC1 BALB/c mice. Cells treated with increasing concentrations of HPB-CD for 2 hours were assessed for cell viability using an MTT assay [13]. Neither cell line shows sensitivity to HPB-CD at concentrations up to 6% (Fig. 3). The NPC1 cells (Fig. 3, circles) begin to exhibit decreased survival at 8% HPB-CD (P<0.01), whereas Wt cells (Fig. 3, squares) are completely resistant up to 10% HPB-CD (P<0.05), suggesting that NPC1 cells are more sensitive to the compound than Wt cells at higher concentrations. All subsequent acute studies were performed using 2% HPB-CD to ensure the lowest cellular toxicity.

**Figure 4:**
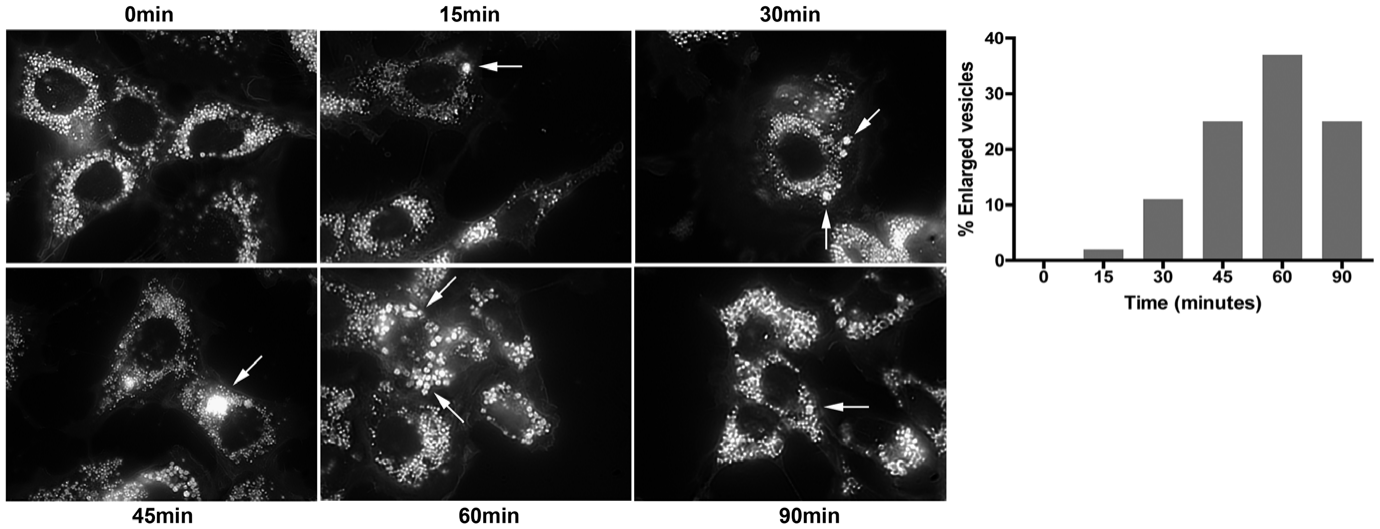
Enlarged cholesterol-filled vesicles in NPC1 cells treated with HPB-CD. Cholesterol-filled vesicles fuse to form larger vesicles (arrows), the number of which increases with time. The bar graph represents the percentage of cells with at least one enlarged vesicle (with a minimum size of 10 microns) out of 150 cells and is representative of 2 independent experiments.

To determine the effects of acute HPB-CD exposure at the cellular level, NPC1 cells were treated with 2% HPB-CD for up to 90 minutes and stained with filipin. As expected, untreated NPC1 cells contain a profusion of vesicles that are filled with free cholesterol (Fig. 4, 0 min). Interestingly, at subsequent time points, larger bright vesicles begin to appear (Fig. 4, 15–60 min, arrow). Cells that contain these larger vesicles do not stain as brightly as their surrounding cells, suggesting that cholesterol may be concentrating in these larger vesicular structures within the cell. The number and size of these larger vesicles increases with time, but by 90 minutes, the number of these structures has decreased (Fig. 4, graph). Taken together, these results suggest that HPB-CD causes cholesterol to concentrate in large vesicular structures, which is consistent with the early stages of lysosomal exocytosis, in which larger vesicles form via vesicle fusion before they are extruded from the cell [14].

### Exposure to HPB-CD induces the secretion of endosomal/lysosomal proteins from cells in a calcium-enhanced manner

A single dose of HPB-CD has been shown to dramatically reduce the unesterified cholesterol pool in NPC1 mice as early as 24 hours after administration, with a concomitant increase in cholesteryl ester formation and fecal bile acid excretion [8], [15]. The speed of this response led us to hypothesize that cholesterol exits cells en masse via lysosomal exocytosis. In cultured cells, administration of the calcium ionophore ionomycin has been shown to stimulate lysosomal exocytosis within minutes by elevating intracellular calcium levels [16], [17].

**Figure 5:**
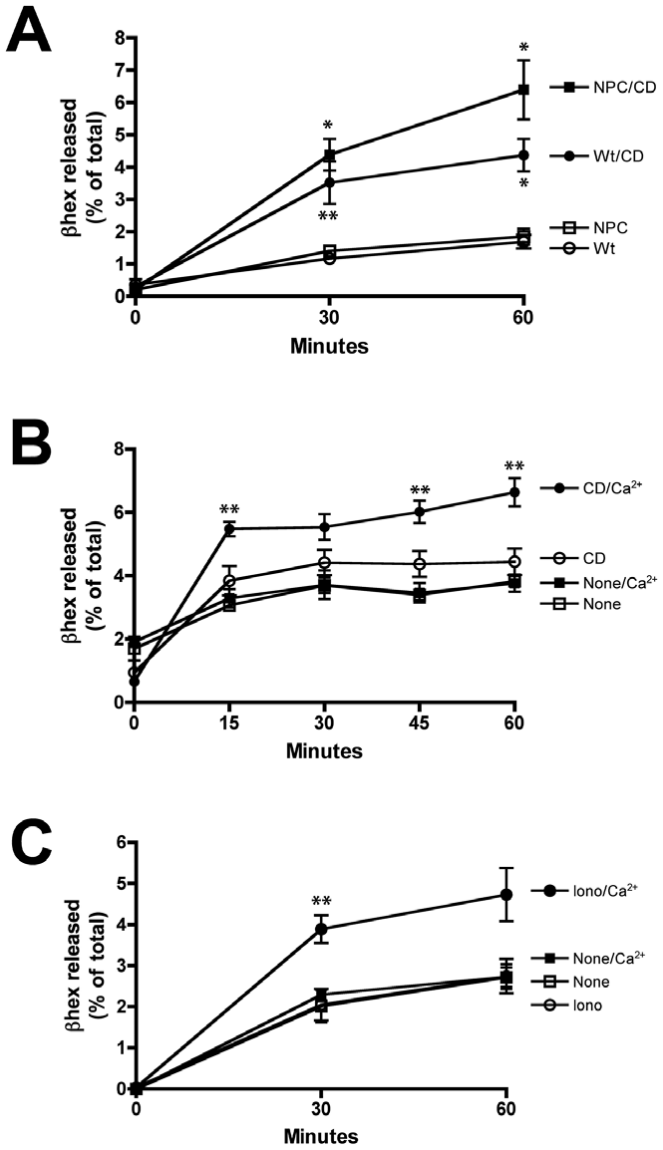
Secretion of the lysosomal enzyme β-hexosaminidase by HPB-CD-treated cells. (A) Wt (circles) and NPC1 (squares) cells were incubated with (closed shapes) or without (open shapes) 2% HPB-CD. At the indicated times, an aliquot of media was assayed for β-hexosaminidase activity. (B) Wt cells were incubated with 2% HPB-CD in the presence (closed shapes) or absence (open shapes) of calcium and an aliquot of media was assayed for β-hexosaminidase activity. (C) Wt cells were incubated with 1 µM ionomycin for 20 min in the presence (closed shapes) or absence (open shapes) of calcium and an aliquot of media was assayed for β-hexosaminidase activity. All enzyme activities are expressed as the percentage of the total enzyme activity found in media and cells. * and ** denote statistically significant differences between treated and untreated cells with *P*<0.01 and *P*<0.05, respectively.

To determine if cyclodextrin, similarly to ionomycin, induces lysosomal exocytosis, the culture media of Wt and NPC1 cells were analyzed for the presence of the lysosomal enzyme β-hexosaminidase (β-hex) as a marker for lysosomal content secretion. In both cell types, there is a significant increase in β-hex activity in the culture media of treated cells compared to untreated cells after 30 minutes (Fig. 5A, closed circles and squares). Furthermore, the level of enzyme activity in the media of treated NPC cells is slightly higher than that found in Wt cells after 60 minutes (Fig. 5A, closed squares), which suggests that NPC cells may be slightly more sensitive to HPB-CD than Wt cells. The appearance of β-hex in the culture media of HPB-CD-treated cells is not a result of generalized cell lysis, since the levels of lactate dehydrogenase in the media remained unchanged in both cells types for the duration of the assay (data not shown).

To evaluate the effect of calcium on HPB-CD-induced β-hex secretion, Wt cells were treated with HPB-CD in the presence and absence of calcium. In untreated cells in the presence and absence of calcium, there is a small percentage of enzyme in the media, which did not increase significantly over time (Fig. 5B, None, *P*>0.1). In contrast, the media from cells treated with HPB-CD in the presence of calcium contain increasing levels of β-hex over time (Fig. 5B, CD/Ca^2+^), and furthermore, these levels are higher than the levels found in the media of cells treated in the absence of calcium (Fig. 5B, CD). These studies were performed in the presence of mannose 6-phosphate to prevent reuptake of β-hex via the plasma membrane mannose 6-phosphate receptor. These results indicate that the induction of β-hex secretion from HPB-CD-treated cells is enhanced by the presence of extracellular calcium. Comparable results were obtained with ionomycin, which affects the release of intracellular calcium stores and the influx of extracellular calcium [18]. Similar to published reports [17], in the absence of calcium, ionomycin has little effect on β-hex secretion (Fig. 5C, Iono), whereas in the presence of calcium, there is a 2-fold increase in enzyme found in the culture media (Fig. 5C, Iono/Ca^2+^).

**Figure 6:**
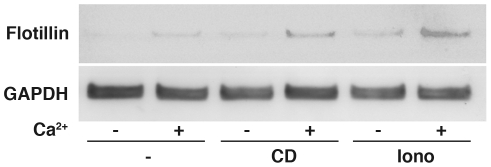
Secretion of exosomes in HPB-CD-treated cells. Wt cells incubated with PBS (−), 2% HPB-CD (CD), or 1 µM ionomycin (Iono) show differential secretion of exosomes in the presence (+) or absence (−) of calcium, as determined by the presence of flotillin in the culture media. Equal loading was determined by normalizing to GAPDH levels in the cell lysate.

Treatment with HPB-CD also stimulated the secretion of flotillin 2, a protein associated with lipid rafts and endolysosomes [19] and a marker for exosomes [20]. These endolysosomal-derived vesicles are released in a calcium dependent manner into the extracellular milieu upon fusion of multivesicular bodies with the plasma membrane [14], [21]. Exosomes are normally secreted by most cells and have a number of functions, including a role in intercellular communication [22]. In untreated cells, low levels of flotillin can be detected in both the presence and absence of calcium (Fig. 6, -), although there is slightly more protein in the calcium-positive sample. Treatment with HPB-CD in the absence of calcium has no effect on exosome secretion compared to untreated cells (Fig. 6, CD−) but in the presence of calcium the levels of flotillin increase (Fig. 6 CD+), indicating an increase in exosome release. This effect of HPB-CD on exosome secretion is similar to, albeit not as potent as, that of ionomycin (Fig. 6, Iono+), which is well known to greatly stimulate exosome secretion [23]. Taken together, these results indicate that HPB-CD treatment induces a rapid, calcium-dependent endolysosomal secretion.

### Endosomal proteins relocalize to the plasma membrane following HPD-CD treatment

**Figure 7:**
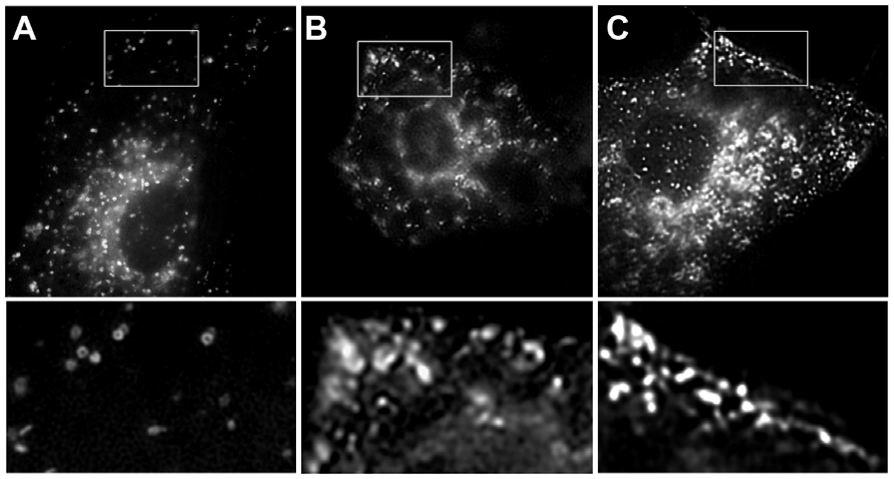
Relocalization of endolysosomal membrane proteins in HPB-CD-treated cells. Wt cells expressing NPC1-GFP exhibit staining in endolysosomal vesicles throughout the cell (A). Following treatment with 2% HPB-CD for 30 minutes, the majority of transfected cells contain protein that is found in vesicles lining the cell periphery (B). This location mimics the position of NPC1-GFP protein found in cells treated with 1 µM ionomycin, which is known to induce endolysosomal exocytosis (C). Greater than 60% of transfected cells in each sample exhibited the phenotypes described.

To determine if the NPC1 compartment participates in exocytosis following HPD-CD treatment, a GFP-tagged NPC1 protein was transiently expressed in Wt cells. As expected, transfected cells show the tagged protein being targeted to endolysosomal vesicles scattered throughout the cell (Fig. 7A). However, after exposure to HPB-CD, a majority of the transfected cells (>60%) contain NPC-positive vesicles that have relocalized near the plasma membrane, presumably prior to fusion with the membrane (Fig. 7B and inset). In support of this notion, similar relocalization of NPC1-containing vesicles is observed after treatment with ionomycin (Fig. 7C and inset), a compound known to induce this type of vesicle relocation as discussed above [17]. These studies provide compelling evidence that endolysosomal constituents are targeted for cellular exocytosis after HPB-CD treatment.

## Discussion

Very little has previously been known regarding the mechanisms of HPB-CD action. The ability of HPB-CD to improve disease progression in NPC1 mice [6], [15] has prompted its recent approval for treatment of NPC1 patients [5]. Although CDs have not generally been considered toxic [24], one recent study showed that several CDs promote the toxic aggregation of beta-amyloid *in vitro* [25]. Such observations make the careful elucidation of CD's actions critical for accurate assessment of long-term outcomes.

Because HPB-CD can bind and extract cholesterol from membranes, it has generally been assumed that this is the mechanism by which HPB-CD clears cholesterol from NPC1 cells. However, there are several questions to arise from this scenario. First, the accumulated cholesterol in NPC1 cells is located in endolysosomal vesicles, and it has never been established that the hydrophilic HPB-CD can cross the plasma membrane and/or organellar membranes. One recent study suggests that cyclodextrins are endocytosed; however, the cyclodextrins in that study were coupled to dextran [26], which is known to enter the endolysosomal system via endocytosis [27]. Furthermore, assuming that HPB-CD can be endocytosed and reach the appropriate compartment to complex with cholesterol, it is also not clear how these HPB-CD-cholesterol complexes can then exit these compartments and redistribute cholesterol to other cellular pools. Finally, several studies indicate that HPB-CD is unable to cross the blood-brain barrier [6], [28], [29], so the mechanism by which HPB-CD improves the neurological symptoms in NPC1 mice [27] remains a great open question.

If CDs extract lipids from cellular membranes as is currently accepted [1], [3], one possible consequence would be the permeabilization of those membranes to small ions such as calcium. Indeed, CDs have been shown to create “holes” in human and simian immunodeficiency virus membranes as well as in artificial lipid bilayers [4], [30]. Microdisruptions in the plasma membrane are normally repaired in cells by lysosomal exocytosis, during which endolysosomes fuse with the plasma membrane [31]. The resealing process is calcium-dependent and results in the extrusion of lysosomal contents from the cell [31]. Because the large structures that are observed after acute exposure to HPB-CD (Figure 4) are not visible prior to HPB-CD treatment, we hypothesize that they are formed through fusion of smaller vesicles prior to extrusion from the cell. However, it is possible that they may be formed via alternate mechanisms or that they may be pre-existing structures into which cholesterol is shuttled after HPB-CD treatment and thus appear positive by filipin staining. The release of β-hex and flotillin, but not lactate dehydrogenase, into the media of HPB-CD-treated cells is consistent with the specific release of endolysosomal contents in a calcium-enhanced manner. Furthermore, a recent study showing that cholesterol can exit cells via exosomes [32] supports our hypothesis that the mechanism by which HPB-CD induces the “emptying” of cholesterol and presumably other lipids from NPC1 cells is lysosomal exocytosis.

The cellular events by which this process is induced remain unclear. Our studies suggest that stress-related signaling pathways are not involved (Figure 1). As discussed above, damage to the plasma membrane may be the trigger that initiates the process. It is also possible that HPB-CD activates other signaling pathways, as has recently been described for cell migration during leukocyte chemotaxis [33]. The observation that lysosomal exocytosis plays such an important role in chemotaxis is relevant to these studies, since it indicates that exocytosis can occur for prolonged periods of time and not only in response to acute injury of the plasma membrane. The lipid accumulation in NPC disease is so massive that short exposure times to HPB-CD produce no discernible decreases in free cholesterol levels (data not shown), even as lysosomal enzymes are being secreted into the media. However, longer exposure times (>20 hours) produce a significant decrease in free cholesterol levels (Figure 2), suggesting that a prolonged dose of HPB-CD is necessary to effect clearance of stored intracellular lipids.

These findings should provide insight into the mechanism of CD action in the treatment of disease. For disorders such as NPC, in which there is accumulation in the endolysosomal system, it may not be necessary to receive continuous doses of HPB-CD since the accumulation of lipids occurs over time. With respect to the therapeutic effects of CD on NPC disease, many questions remain, such as what happens to the lipids following lysosomal exocytosis? Does CD cause relocalization of lipids to the plasma membrane via exocytosis and reabsorption, or do neighboring cells take up and process/digest these regurgitated lipids? Further studies are necessary to determine the exact *in vivo* mechanism of CD action. Finally, caution should be exercised in the interpretation of experimental data involving the use of CD to extract or manipulate plasma membrane cholesterol since these studies indicate that CDs have pleiotropic effects on cellular physiology.

## Materials and Methods

### Materials

Pharmaceutical grade 2-Hydroxypropyl- β-cyclodextrin (HPB-CD; trade name Trappsol) was a kind gift of CTD, Inc. Chemicals were from Sigma-Aldrich (Milwaukee, WI) unless otherwise indicated. Filipin was from Polysciences Inc., (Warrington, PA). OPTIMEM and Lysosensor Green-189 were from Invitrogen (Carlsbad, CA). FuGENE™ 6 transfection reagent was from Roche Diagnostics. The ERK and JNK antibodies were from Cell Signaling Technology (Beverly, MA). The GAPDH antibody was from Millipore (Billerica, MA) and the flotillin 2 antibody was from BD Biosciences (San Jose, CA).

### Cell lines, transfection, and microscopy

We have previously described the establishment and maintenance of Wt and NPC1 cell lines derived from BALB/c mice [34]. Cells were treated with 2% HPB-CD in OPTIMEM in experiments except where otherwise indicated.

For filipin staining, cells were incubated with HPB-CD and then stained with filipin as we have previously described [35]. Fluorescence was analyzed using a Nikon Eclipse microscope fitted with a charge-coupled-device camera. Images were acquired with MetaVue software using the same exposure time for all samples and then deconvoluted using AutoDeblur software. Fluorescence intensity was determined using the integrated intensity function of MetaVue software; at least 150 cells were quantitated for each sample and each experiment was repeated 3 times.

Transient overexpression of the NPC1 protein fused to GFP (pGS-NPC1-GFP) was carried out by transfecting cells at 60% confluency using FuGENE 6 reagent according to the manufacturer's recommendations. At 24 h post-transfection, cells were treated with HPB-CD for 15 min or 1 µM ionomycin for 30 min before live cells were mounted onto glass slides and analyzed for NPC1-GFP expression.

### Western blot analyses

For ERK/JNK westerns, cells were incubated with HPB-CD for the indicated times and then lysed in buffer (150 mM NaCl, 50 mM Na_2_HPO_4_, pH 6.9, 1 mM EDTA, 1% Igepal) for 5 min at 4°C. Lysates were clarified by centrifugation at 20,000 *g* for 5 min and protein concentrations were determined using the fluorescamine method as we have described [36]. Between 15–30 mg of protein was subjected to electrophoresis through a 4–12% Bis-Tris pre-cast gel (Invitrogen, Carlsbad, CA, U.S.A.) in MES buffer and transferred on to a Protran membrane as we have described [34]. Proteins were visualized by chemiluminescence using SuperSignal West Dura substrate (Thermo Scientific, Rockford, IL) on a FluorChemQ imager using AlphaView software (Alpha Innotech, San Leandro, CA).

For exosome isolation, cells were incubated with HPB-CD or 1 µM ionomycin in PBS for 30 minutes at 37°C. The media was collected and exosomes isolated by centrifugation at 100,000 *g* for 1 hr. Exosomes were resuspended in 20 mM Tris-HCl, pH 8.0 and sonicated 3×5 minutes in a bath sonicator. Cells were lysed and cellular protein concentrations determined as described above. Exosome loading was normalized to the cell lysate protein concentrations.

### MTT Assays

Cells were seeded at 15,000 cells/well in a 96-well plate and incubated overnight at 37°C. The next day they were incubated with HPB-CD for 2 hr at 37°C, and cell viability was assessed by an MTT assay as described [37].

### β-hexosaminidase assays

Cells were incubated with HPB-CD or 1 µM ionomycin and 2 mM mannose-6-phosphate at 37°C. For calcium studies, cells were incubated with the appropriate compounds in PBS and 1.2 mM CaCl_2_ was supplemented for the calcium-positive samples. At the indicated time points, an aliquot of media was removed and assayed for β-hex activity at 37°C and pH 4.4 by using the synthetic substrate 4-methylumbelliferyl-N-acetyl-glucosaminide as has been previously described [34]. After the last time point, cells were lysed as described above and an aliquot of the lysate assayed for β-hex activity to determine the total enzyme activity of each sample. Enzyme activities are expressed as a percentage of the total enzyme activity found in the media and lysate.
